# Structure Modulates Similarity-Based Interference in Sluicing: An Eye Tracking study

**DOI:** 10.3389/fpsyg.2015.01839

**Published:** 2015-12-18

**Authors:** Jesse A. Harris

**Affiliations:** Department of Linguistics, University of California, Los AngelesLos Angeles, CA, USA

**Keywords:** working memory, similarity-based interference, ellipsis, eye tracking, sentence processing

## Abstract

In cue-based content-addressable approaches to memory, a target and its competitors are retrieved in parallel from memory via a fast, associative cue-matching procedure under a severely limited focus of attention. Such a parallel matching procedure could in principle ignore the serial order or hierarchical structure characteristic of linguistic relations. I present an eye tracking while reading experiment that investigates whether the sentential position of a potential antecedent modulates the strength of similarity-based interference, a well-studied effect in which increased similarity in features between a target and its competitors results in slower and less accurate retrieval overall. The manipulation trades on an independently established Locality bias in sluiced structures to associate a *wh*-remnant (*which ones*) in clausal ellipsis with the most local correlate (*some wines*), as in *The tourists enjoyed some wines, but I don't know which ones*. The findings generally support cue-based parsing models of sentence processing that are subject to similarity-based interference in retrieval, and provide additional support to the growing body of evidence that retrieval is sensitive to both the structural position of a target antecedent and its competitors, and the specificity or diagnosticity of retrieval cues.

## Introduction

The rapid formation of non-adjacent syntactic dependencies during online sentence comprehension offers intuitive evidence for the importance of an efficient retrieval system. Two well-studied cases are argument-verb dependencies, in which an argument must be related to its verb no matter the amount of intervening material (1a), and anaphoric dependencies, in which a pronominal element, like *him* or *one*, is associated with another co-referring expression, possibly from among multiple possibilities (1b). The noun phrase *the barber* must be retrieved from memory in each case: either in the subject-trace (gap) position *t*_1_ (1a) or else as a co-referring expression (1b). Only in the former case is the dependency unambiguously determined by structure; the second case is simply the most plausible given the topicality of *the barber* and real world knowledge, as illustrated by fact that likely co-reference possibilities depend on the predicate (1c).

(1)
  - The barber_1_ (that John_2_ wanted his son_3_ to visit) t_1∕^*^2,^*^3_ died yesterday.
  - He_1>2>3_ was 95 years old.
  - He_2>3,#1_ was saddened to hear the news.

Findings from both types of cases support recent cue-based parsing models of sentence processing in which all possible antecedents are activated in parallel through a fast, domain-general associative cue-matching procedure (for review of evidence and models see, e.g., Lewis et al., 2006; Van Dyke and Johns, 2012; Caplan and Waters, 2013). In such models, a retrieval cue—e.g., the verb *died* in (1a) or the pronoun *he* in (1b), initiates direct access to all possible targets (possible antecedents or dependencies) from memory. However, not all possible targets must be equally activated within memory: some targets might receive greater activation by virtue of sharing syntactic or semantic features with the retrieval cue, while other targets might receive less activation as a function of temporal decay (Van Dyke and Johns, 2012, for review, though a reviewer points out that decay as the primary source of forgetting is not strongly supported in the general memory literature, as demonstrated by Keppel and Underwood, 1962 and Waugh and Norman, 1965). Further, the allocation of attentional resources in memory is severely constrained, possibly limited to a single item within the focus of attention (e.g., McElree, 2001). Thus, the memory architecture employed in sentence processing strongly resembles the architecture thought to underlie domain-general tasks (e.g., McElree, 2000; McElree et al., 2003; Van Dyke and Lewis, 2003; Lewis and Vasishth, 2005; Lewis et al., 2006).

A major research question within the cue-based parsing literature addresses the extent to which domain-specific knowledge influences the retrieval process; in particular, whether syntactic constraints affect antecedent retrieval, and, if so, at precisely what stage of retrieval. Much of the research conducted thus far has examined whether syntactically inaccessible targets are considered viable candidates for retrieval. The two major schools of thought addressing this issue in the cue-based parsing literature are (i) structure-based accounts, and (ii) unconstrained-cue accounts. In the former, grammatical constraints filter the set of possible antecedents retrieved from memory by constraining the search set to grammatically permissible positions (e.g., Nicol and Swinney, 1989; Sturt, 2003; Xiang et al., 2009; Chow et al., 2014). Thus, items in structurally inaccessible positions would be effectively ignored in the retrieval process, though grammatically illicit items may feed repair processes triggered by retrieval failure (Chow et al., 2014). In contrast, the latter, unconstrained-cue, approach proposes that grammatical constraints are but one of the many possible factors guiding retrieval (e.g., Badecker and Straub, 2002; Lewis and Vasishth, 2005; Chen et al., 2012; Jäger et al., 2015). Badecker and Straub (2002), for example, propose that the set of possible antecedents are restricted not by tree geometry, but a number of other factors, such as attention or discourse importance. Elements in the focus of attention are often identified with discourse topics or a center, though details regarding how to define topics vary considerably (see, e.g., Chafe, 1976; Gundel, 1985; Gundel et al., 1993; Grosz et al., 1995). Under this type of account, syntactically inaccessible items might well interfere with retrieving a target, particularly if such items are highly salient in the discourse, though, of course, structure may be one of the factors that determines discourse salience. Although it should be clear that both viewpoints agree that structural information is a factor in retrieval, they permit very different types of mechanisms by which structure is utilized.

Unfortunately, results that would clearly arbitrate between the two camps are somewhat mixed. For example, while filler-gap dependencies are sensitive to syntactic islands (for review see, e.g., Sprouse et al., 2012), retrieving pronouns and reflexives has sometimes been found to be susceptible to interference from structurally inaccessible items (e.g., Badecker and Straub, 2002; Jäger et al., 2015; though see Chow et al., 2014 for a failure to replicate Badecker and Straub's results). The general finding is that syntactic information plays an important role in retrieval for at least certain types of dependencies (Van Dyke and McElree, 2006, 2011; Van Dyke, 2007; Dillon et al., 2014; Cunnings et al., 2015), even if it is currently unclear whether that role is to filter possible antecedents or provide weighted or probabilistic cues regarding their likelihood of matching the probe (Badecker and Straub, 2002; Cunnings et al., 2015). Further, it is unclear at this stage whether syntactic information should be treated as value in a feature bundle (e.g., Lewis and Vasishth, 2005), and if configurational relations like c-command inherently preclude such a treatment (see Alcocer and Phillips, 2012; Kush, 2013, for discussion).

The present research seeks to widen the empirical coverage of the issue of structural import by leveraging an independently established bias guiding the resolution of correlate-remnant pairs in clausal ellipsis (sluiced) structures. In (2), for example, the remnant *which (ones)* might be paired with either a subject correlate (*a few linguists*) or an object correlate (*some silly examples*), spelled out as (2a) and (2b), respectively.

(2) [A few linguists] gave [some silly examples], but I don't remember which (ones).
  - …I don't remember which linguists. (Subject correlate)
  - …I don't remember which examples. (Object correlate)

Although sluicing ellipsis permits correlates in both subject and object position above, it shows a strong preference for the latter (Frazier and Clifton, 1998). As mentioned, much research in content-addressable retrieval systems in language processing addresses whether grammatical restrictions govern the resolution of various types of anaphoric dependencies, especially the availability of syntactically illicit antecedents (e.g., Sturt, 2003; Martin et al., 2012, 2014; Cunnings et al., 2015; see also Phillips et al., 2011, for review). In contrast, correlates to sluiced remnants are merely heavily biased, rather than constrained grammatically. Thus, sluiced structures provide a potentially revealing counterpoint to studies investigating syntactic barriers to accessibility: if structurally defined preferences, in addition to grammatical restrictions, influence the retrieval process, the retrieval system may use structural information to privilege some products of memory over others. In which case, the retrieval system might be said to avail itself of domain-specific, linguistically defined structural preferences, in addition to hard-coded grammatical principles. In the remainder of the paper, I review the aspects of cue-based parsing that are most relevant for this study, along with the basic assumptions regarding the ellipsis structures explored here. I then present two experiments which collectively support the idea that retrieving antecedents in sluiced sentences is subject to interference effects (congruent with Martin and McElree, 2011, discussed in detail below) and that the strength of such interference effects depends on the sentential position of possible targets and strength of the cue provided at retrieval.

### Cue-based parsing and similarity-based interference

Many models of how linguistic representations are encoded and retrieved during real-time language comprehension have been proposed (for review, see Van Dyke and Johns, 2012; Caplan and Waters, 2013). In contrast to cue-based retrieval systems, traditional models employ an operation that searches through items in memory, typically in a serial fashion (Dosher and McElree, 1992; McElree and Dosher, 1993), as developed for short-term memory in general (Sternberg, 1966, 1975). In these models, the path of the search varies according to whether the search queue starts with the first item encountered, as in forward search, or the most recent item, as in backward search. The basic prediction of search models is that retrieval time should increase as a function of search space within the path: the more items that must be searched through, the greater the search time required to do so. This prediction, however, has not been supported by Speed Accuracy Tradeoff (SAT) experiments which report that speed of reaching a stable judgment or interpretation is unaffected by the size of the putative search space, even though accuracy is (McElree and Dosher, 1989; McElree, 2000, 2006; McElree et al., 2003; Foraker and McElree, 2007, 2011; Martin and McElree, 2008, 2009, 2011). For example, McElree (2001) manipulated the amount of intervening material (underlined) between a relative clause filler *the book* and its object position gap *t*_1_, shown in (3). Subjects were trained to make an acceptability judgment in response to tones at various post-sentence intervals. Measuring the rate at which responses achieved a stable interpretation (the asymptote) as a function of time, he found that while accuracy decreased as more material intervened between the filler and the gap, the speed at which the asymptote was reached did not (see also McElree et al., 2003).

(3)
  - This was [the book]_1_ that the editor admired *t*_1_.
  - This was [the book]_1_ that the editor who the receptionist married admired *t*_1_.
  - This was [the book]_1_ that the editor who the receptionist who quit admired *t*_1_ married.

That accuracy, not the rate of reaching the asymptote, is affected by increasing items in memory not only provides an important argument against search models, it also suggests that the quality of the representations recovered is susceptible to interference from distractors. A content-addressable retrieval system is able to account for this tradeoff by proposing that all items are compared against the target in parallel, not by a search operation, but by an automatic associative cue-matching procedure which compares partial representations of items in memory (possibly encoded as bundles of semantic and grammatical features; Clark and Gronlund, 1996) against cues from the target (as in ACT-R models, Anderson et al., 2004; Lewis et al., 2006). Thus, linear or structural distance is irrelevant for retrieval times, but additional competitors interfere with the cue-matching process by introducing partial matches, thereby degrading the quality of the retrieved item. In other words, the greater the similarity or overlap between a target and its competitors, the greater the effect of interference (Crowder, 1976; Anderson and Neely, 1996).

Similarity-based interference, i.e., the failure to successfully distinguish a target from similar competitors in retrieval, has now been well documented both in general memory manipulations (Nairne, 2002; Öztekin and McElree, 2007) and in language processing contexts (Lewis, 1996; Gordon et al., 2001, 2002, 2004, 2006; Van Dyke and Lewis, 2003; Van Dyke and McElree, 2006, 2011; Van Dyke, 2011; Autry and Levine, 2014, among others). Dual task paradigms combine the two, so that a subject attempts to retain a list of words in working memory while processing a separate sentence for comprehension. When items in the memory set are similar to critical words in the sentence, performance decreases on both reading speed and comprehension accuracy (Fedorenko et al., 2006), especially if those items overlap with retrieval cues for long distance dependencies (Van Dyke and McElree, 2006).

What is less clear is the extent to which structural information modulates the accessibility of a target. On the one hand, grammatically illicit cues appear to license a syntactically dependent element in case of illusory licensing (including negative polarity items, NPI, Vasishth et al., 2008; Xiang et al., 2013, as well as agreement attraction, Wagers et al., 2009; Dillon et al., 2013). To illustrate, comprehenders often accept configurations in which an NPI licensor like *no* simply precedes, rather than c-commands, the NPI *ever*, as in *The restaurants that no newspapers have recommended in their reviews have ever gone out of business*, raising the possibility that configurational information may sometimes be ignored. On the other hand, several recent studies show that distractors within the same syntactic position more greatly interfere in the formation of long distance dependencies (Van Dyke and McElree, 2006, 2011; Dillon et al., 2013, 2014). For example, Van Dyke and McElree (2011) observed that syntactic constraints limit the amount of interference exerted by semantically similar distractors, without eliminating interference completely (also Van Dyke, 2007; Van Dyke and Johns, 2012). They attribute these results to greater disturbance from *retroactive interference*, in which retrieval is hampered by a distractor D that separates a cue C from its target T (schematically: T-**D**-C), than from *proactive interference*, in which retrieval is impeded by distractors processed before the target (**D**-T-C).

Similarly, Dillon and colleagues find an increased interference effect for structurally distant antecedents of a reflexive *ziji* in Mandarin Chinese—i.e., when a distractor intervened between a target and the reflexive. They propose that structurally local domains restrict the initial area for dependency formation.

(4) **Local search hypothesis:** The parser uses positional syntactic information during the retrieval of syntactic dependents, and positional cues serve to restrict retrieval to constituents in some local syntactic domain.

The Local Search Hypothesis contrasts with content-addressable retrieval models that would explain putative effects of Locality in terms of activation decay (see comments in Lewis et al., 2006). Lewis and Vasishth (2005), for example, sharply discriminate items within the focus of attention from those outside of it, which are subject to decay, unless they are reactivated during retrieval processes. A model of this type need not rely on structural information, or even serial order, for retrieval cues. To the extent that syntactic information is utilized, it is established through encoding of morphosyntactic features like [±Theme] or [±Object] in their feature bundles, which collectively identify appropriate structures for retrieval. Thus, such models are entirely domain-general in the sense that the memory operations active during retrieval of linguistic material are the same as those that are active during other types of retrieval. If correct, this uniformity would be a powerful virtue—why postulate specialized retrieval procedures for linguistic structures when the unique aspects of language parsing could be captured simply in terms of specialized features comprising the representations that form the products of memory? In other words, what would be distinctive about language processing would be not so much the *mechanisms* involved in retrieval as how objects over which such mechanisms operate would be *encoded* in memory.

Nevertheless, the following study lends further support to the general finding that the sentence position for an antecedent is relevant during the retrieval process as linguistic dependencies are resolved, though it cannot by itself resolve whether it is best to conceive of such information as structural in nature over sequential or temporal orderings. The study capitalizes on key properties of sluicing ellipsis, as introduced in the following section.

### Sluicing and the locality bias

Sluicing describes focus-sensitive clausal ellipsis after a *wh*-question (Ross, 1967, 1969; Chung et al., 1995; Merchant, 2001, among others), such as (5a) below. Following Merchant (2001) and others, I assume an account of sluicing which derives the overt structure through movement of the *wh*-element *who*_1_ from its base-generated, clause-internal position to a fronted position followed by optional ellipsis <he is meeting t_1_> of the remainder of the clause. Thus, the unelided sentence (5b) is the source for the sluice (5a).

(5)
  - John is meeting {someone/a friend} for dinner, but I can't tell you who_1_ <he is meeting t_1_>.
  - John is meeting {someone/a friend} for dinner, but I can't tell you who_1_ he is meeting t_1_.

Sluicing places restrictions on the types of nouns that can serve as correlate to the remnant, though these restrictions depend on the type of *wh*-element and its restrictor residing in the remnant (Chung et al., 1995; Romero, 1998). For example, proper names and definite nouns are often unacceptable correlates for a *who*-remnant unless it is followed by *else* (6a). In select cases, the *wh*-element may co-refer with an adjunct (6b) or argument (6c) correlate that did not appear in the antecedent clause overtly, in an operation that Chung et al. (1995) call “sprouting.”

(6)
  - John is meeting Mary/the president for dinner, but I can't tell you who ^*^(else).
  - John is meeting Mary/the president for dinner, but I can't tell you where/why/with who.
  - John ate, but I can't tell you what_1_.

Sluicing, along with other forms of ellipsis, has received much attention in recent processing literature (Frazier and Clifton, 1998, 2005, 2011; Carlson et al., 2009; Martin, 2010; Poirier et al., 2010; Dickey and Bunger, 2011; Martin and McElree, 2011, among others). Previous results from processing ellipsis support the expectations of content-addressable retrieval systems, in that retrieval of antecedent material at the ellipsis site appears not to be affected by the size or complexity of the recovered material, an effect explained as either a cost-free copying mechanism (Frazier and Clifton, 2001, 2005; Frazier, 2008) or as a direct pointer in memory (Martin and McElree, 2009). In addition, Martin and McElree (2011) find that increasing the distance to a correlate in sluiced sentences affects retrieval accuracy, not retrieval speed, as predicted by content-addressable systems in which retrieval speed is held constant (as various models of retrieval propose, e.g., McElree, 2000; McElree et al., 2003; Lewis and Vasishth, 2005; Lewis et al., 2006). More detailed comparison to previous studies on sluiced sentences and cue-based parsing is delayed until the General Discussion.

Another important general result is that sluices show a structural preference to associate the remnant with the most local correlate in the antecedent clause, a principle formalized as the Locality bias below (see also Harris and Carlson, 2015, for a similar preference with *let alone* ellipsis).

(7) **Locality bias:** Associate the remnant of clausal ellipsis with a correlate occupying the structurally most local position.

Initial evidence for the Locality bias came from Frazier and Clifton (1998), who manipulated whether a sluiced sentence contained one or more possible correlates (8). In a self-paced reading study, they found that cases with multiple possible antecedents (8b) were read faster than unambiguous structures (8a). The penalty for (8a) can be attributed, in effect, to a violating the preference for Local correlates^1^.

(8)
  - Somebody claimed that the president fired someone but nobody knows who.
  - Somebody claimed that the president fired Fred but nobody knows who.

Carlson et al. (2009) provide additional support for the Locality bias for sluiced sentences in an auditory questionnaire. They observed that unless the subject was focus-marked by a pitch accent or an *it*-cleft, sentences with object correlates were rated higher than alternatives.

A similar Locality bias has been observed for sluices with sprouted antecedents. Frazier and Clifton (2005) report a naturalness rating and reading time advantage when a verb with an implicit object (*studied*) appeared in second conjunct position (*slept and studied*) as compared to first conjunct position (*studied and slept*), as in *Michael (slept and studied*/*studied and slept), but he didn't tell me what*^2^. The basic result coheres with the expectations of the Locality bias in that near antecedents confer a processing advantage over far antecedents (see also Martin and McElree, 2011), though it does complicate the prediction that antecedent distance or complexity is not relevant to retrieval. As an aside, the Locality bias is not unique to sluiced sentences. It has been observed in other move-and-delete types of ellipsis, such replacives (Carlson, 2013) and *let alone* ellipsis (Harris and Carlson, 2015), as well as ambiguous gapping structures (Carlson et al., 2005).

The locus of the Locality bias is open to multiple possibilities. One such possibility derives from the assumption that the processor must ultimately recover elided material for interpretation, presumably by employing default biases and cues from information structure. Another, perhaps not mutually exclusive, possibility suggested by Frazier and Clifton (1998) and Carlson et al. (2005, 2009) is that the most likely correlate is determined by default-focus marking on the most embedded constituent (Selkirk, 1984; Cinque, 1993). In canonical English SVO sentences, the most deeply embedded constituent happens to be the object. For whatever reason, the preferences guiding remnant resolution in sluicing ellipsis appear to diverge from the first-mention bias established for third person pronouns, in which a pronoun is preferentially associated with the subject of a preceding clause (Arnold, 1998, among others).

In any event, there seems to be good evidence that sluiced sentences prefer the most local correlate as the antecedent for the remnant. We now turn to how the expectation for Local correlates might affect content-addressable retrieval systems, as outlined above.

### The current study

An important advantage of using sluicing ellipsis to address the questions above is that the retrieval cues may be explicitly manipulated by modifying the inner restrictor of the *wh*-element. In example (2), for instance, the correlate-remnant pair can be disambiguated simply by repeating the nominal phrase directly, as in *A few linguists gave some silly examples, but I don't remember which linguists*. Such cases determine which noun functions as the correlate to the remnant by completely specifying the relationship: in such cases, there is *total overlap* between remnant and correlate. The eye tracking experiment below exploits this possibility by manipulating whether the restrictor is completely specified by a nominal like *which tourists/wines* (cue-rich probe) or partially specified by a pronoun like *which ones* (cue-poor probe), along with whether the indefinite (assumed to be the preferred correlate) appears in the preferred object location (9a) or not (9b). In addition, it manipulated whether a definite noun distractor appeared in the plural, thereby providing partial cue overlap with the indefinite.

(9)
  - The tourists sampled some wine(s), but I don't know which wines/ones.
  - Some tourist(s) sampled the wines, but I don't know which tourists/ones.

Sluiced sentences like (9) conceivably involve two instances of retrieval: first, the recovery of the elided IP after the remnant, and, second, the pairing between the remnant and the correlate. Regarding the recovery of the elided IP, I adopt an approach in which a syntactic representation is recovered through some sort of cost-free mechanism, such as syntactic copying or recycling (Frazier and Clifton, 2001, 2005) or a pointer in memory (Martin and McElree, 2009), such that the size and complexity of the antecedent clause is essentially irrelevant for retrieval speed (Martin and McElree, 2009, 2011).

Regarding the pairing of the remnant with the correlate, there are several theoretical options to consider, especially with respect to the different types and strengths (diagnosticity) of cues in the remnant. First, we might imagine that the parser forms a dependency between the remnant and correlate selectively, that is, only when the remnant contains a pronoun, as in *ones*, but not when its inner restrictor is fully specified, as in *tourists* or *wines*. In this case, a fully specified restrictor could be interpreted via straightforward composition, without retrieving a correlate. However, this approach is unlikely given results from sprouting in sluicing ellipsis, which show a penalty when there is no overt correlate in antecedent clause (Frazier and Clifton, 1998; Dickey and Bunger, 2011).

The two remaining options would require that a dependency be formed between all types of correlates and remnants, but differ in what type of mechanism establishes it. One option to consider is one in which the nominal in the restrictor obviates the associative cue-matching procedure by forming a direct link to the previous instance of the noun, trivially avoiding cue overload effects altogether. Another option is that establishing a dependency between correlate and remnant evokes an associative cue-matching procedure, as proposed for anaphoric dependencies in general, but mitigates cue overload effects by virtue of the total overlap in cues between the remnant probe and the target correlate. In either case, we would expect the strength of the cue at the remnant to modulate the retrieval process. For concreteness, I adopt the latter approach, acknowledging that the experiments below do not depend on or arbitrate between these two possibilities.

As observed by a reviewer, it may be important that the two types of dependencies are not independent: if a comprehender resolves the remnant to an object-position correlate in (9), she is also committed to a particular syntax for the IP ellipsis, e.g., *[which wines]*_1_ *they sampled t*_1_/*[which tourists]*_1_ *t*_1_ *sampled them*. While this dependency should be explored in depth, we will not do so here^3^. I will simply assume that however recovery of the IP ellipsis impacts retrieval of the remnant, the effects will be comparable across conditions.

An important conceptual issue for cue-based parsing models in general is what types of information constitute cues for retrieval. In such models, it is conceivable that any information coded as a feature value in a feature bundle is qualified to serve as a cue for retrieval, though some types of information, especially relational information, might be less amenable to representation by features than others (Kush, 2013). Following recent literature (e.g., Lewis and Vasishth, 2005), I assume that retrieval is driven, at least in part, by features from lexical (gender and number) and morphosyntactic (grammatical roles and case) information derived from context, and that what is retrieved are partial representations of constituents. Further, retrieval occurs whenever an item has to be associated with another item in memory for complete interpretation, including better-studied cases of anaphora resolution, verbal agreement, NPI licensing, and variable binding, although different kinds of dependencies might attend to distinct types of information. I remain largely agnostic about the internal organization of retrieval with respect to other interpretive processes, e.g., whether retrieval is discrete, continuous, or cascaded, as the study was not designed to address these issues, and the results below are consistent with any number of possibilities.

Assuming an associative cue-matching procedure and a preference for local antecedents, the reading experiment below was designed to test the following two basic predictions:

**P1. Locality:** The most local antecedent, in this case the object noun, is favored for retrieval.
**P2. Nominal Advantage:** Nominal restrictors (*which tourists*/*which wines*) include a rich set of cues specifying retrieval, and thus facilitate retrieval over cue-poor probes like *which ones*.

The most important prediction, however, is one in which distractors outside the local (object) domain are subject to varying degrees of interference; a strong effect of interference is predicted only in case of partial overlap, as fully specifying the inner restrictor with a nominal should eliminate the effects of cue overload, either by delivering the appropriate correlate directly, or via total overlap between the remnant and the target correlate.

**P3. Structure-Dependent Interference:** A retrieval penalty for violating Locality
  - arises when a distractor in the preferred (object) position shares features with the remnant, and
  - increases if retrieval is initiated by cue-poor probes (*ones*).

Prior research investigating the effect of structural constraints on retrieval has often used the gender feature in a feature mismatch paradigm (e.g., Clifton et al., 1999; Badecker and Straub, 2002; Sturt, 2003; Chow et al., 2014). Manipulating gender agreement between the remnant and the antecedents was not possible here, given that English does not encode gender for impersonal pronouns like *ones*. Therefore, we must first show that plural definite nouns are viable correlate competitors for unambiguous *which* remnants, like *which tourists* or *which wines*, the central task of the following experiment. An affirmative finding will support the assumption that the plurality feature sufficiently induces similarity-based interference effects in the formation of correlate-remnant pairs with pronouns like *ones* in the next experiment. In addition, it will address the assumption regarding whether the indefinite determiner *some* marks the preferred correlate for sluices with *which*-remnants as opposed to the definite determiner.

As a final terminological note, the present use of “interference” diverges somewhat from a common use in the literature, in which the distractor is not a grammatical antecedent, or otherwise inaccessible (e.g., Van Dyke, 2007; Phillips et al., 2011). If both nouns in the matrix are acceptable as antecedents, the manipulation might be best cast in terms of a “fan effect” in which multiple non-referents interfere with dependency resolution (Anderson, 1974; Anderson and Reder, 1999; Autry and Levine, 2014). However, as the effects in either case would ideally be driven by the same underlying types of retrieval mechanisms, I retain the use of interference here, in hopes of expanding the empirical range of strongly biased, though not strictly speaking ungrammatical, structural preferences.

## Experiment 1

A forced-choice completion test was first conducted over the Internet in order to determine the extent to which a plural definite noun competes with a plural indefinite as a correlate. I take such cases to be indicative of similarity-based interference effects, although they may differ in kind from other types of interference. A further question is whether the extent to which plurality makes a definite noun an appealing correlate is affected by its structural position.

### Method

#### Participants

Twenty-nine subjects were recruited using Amazon Mechanical Turk, an Internet-based service where individuals complete short tasks online for payment. One subject self-identified as a non-native English speaker, and was removed from analysis. A pretest evaluated subjects' competency with three difficult to interpret questions. Three subjects were removed for answering one or more of these questions incorrectly. Four catch items were included to identify inattentive subjects, but no subject was removed on this basis. However, an additional subject was removed for counterbalancing purposes, leaving 24 subjects in the final data set. All subjects were compensated $4 for their participation, regardless of native language or performance. This experiment, along with the following, were carried out with prior Internal Review Board approval from Pomona College. All subjects gave written informed consent before starting the experiment, and were permitted to remove themselves at any time from the procedure without penalty.

#### Materials

The 2 × 2 experimental design crossed Indefinite Location (Object indefinite, Subject indefinite) with Definite Number (Plural, Singular). The levels of the Indefinite Location condition were determined by its syntactic position in the matrix clause. In other words, there were two sequences of determiner in the matrix clause: either (i) a definite subject (singular or plural) followed by a plural indefinite object, or (ii) a plural indefinite subject followed by a definite object (singular or plural). The Plural condition was created from the Singular condition simply by adding the plural marker to the definite noun phrase, e.g., *tourist* ~ *tourists* or *wine* ~ *wines*. All critical nouns except one (*fireman* ~ *firemen*) were regular plurals. Twenty-four quartet fragments like (10) were constructed below. Items are reported in Appendix A of Supplementary Material.

(10) Object indefinite
  a. *Plural definite:* The tourists sampled some wines, but I've forgotten…
  b. *Singular definite:* The tourist sampled some wines, but I've forgotten…
    Subject indefinite
  c. *Plural definite:* Some tourists sampled the wines, but I've forgotten…
  d. *Singular definite:* Some tourists sampled the wine, but I've forgotten…

Two forced-choice completions (11) were provided under the fragments in (10). The response options always agreed in plurality with the preceding sentence fragment, e.g., *tourists*/*wines* in (10a,c), *tourist*/*wines* in (10b), and *tourists*/*wine* in (10d). Answers were presented in a different random sequence for each subject.

(11) Forced-choice options
  - Subject correlate response: which tourist(s).
  - Object correlate response: which wine(s).

After a short guided practice consisting of three sample sentences, subjects were presented with an additional 52 items from unrelated experiments with various structures, 12 non-experimental fillers, in which both responses were acceptable, and four catch items permitting only a single correct answer, for a total of 92 items.

#### Procedure

Items were presented in an individually randomized and fully counterbalanced order, so that subjects saw one and only one sentence fragment from each quartet. Subjects were instructed to rely on their intuitions to select whichever response would make “the resulting sentence sound the most natural.” Subjects were given an hour to complete the task, but all subjects finished within 40 min, with an average of 25 min per subject. In addition, encrypted versions of IP addresses were recorded to identify subjects who may have taken the experiment more than once. No such cases were observed.

#### Results

One item (item 1 in Appendix A of Supplementary Material) contained a typo and was removed from analysis. Data analysis was conducted in R version 3.1.2 (R Development Core Team, 2014). Mean percent and standard deviations for *subject completion* responses by condition are presented in Table 1.

**Table 1:** **Experiment 1: percent *subject* response selected**.

**Indefinite location**	**Definite number**		**Correlate mean**	**Attraction effect**
	**Plural**	**Singular**		
Object	28% (4)	19% (3)	24% (3)	−9%
Subject	67% (4)	75% (4)	71% (3)	8%

*Standard errors are in parentheses*.

Conditions were given sum (deviation) coding so that the hypothetically simplest condition, the Object Correlate—Singular condition, was treated as the statistical baseline. The response data was modeled as a logistic linear mixed effects regression model using the lme4 package (Bates and Maechler, 2009) with by-subjects and by-items random slopes and intercepts, shown in Table 2.

**Table 2:** **Experiment 1: results of linear mixed effects regression model**.

	**Estimate**	**Std. error**	**Wald *Z***	***p*-estimate**
(Intercept)	−0.179	0.362	−0.494	0.621
Correlate location	**1.661**	**0.285**	**5.834**	< **0.001**
Definite number	−0.038	0.208	−0.183	0.855
Correlate × Definite number	−**0.473**	**0.199**	−**2.376**	< **0.05**

*Significant effects are printed in bold*.

As expected, the choice between Subject and Object correlate response closely corresponded to the location of the indefinite *some*: subject position indefinites garnered greater overall Subject correlate responses (*M* = 71%, *SE* = 3) than object position indefinites (*M* = 24%, *SE* = 3), *z* = 6.77, *p* < 0.001. The result confirms the intuition that language users prefer indefinites as correlates to remnants in sluiced structures, though we should note that the preference is not absolute; see also the Discussion Section of Experiment 2, which acknowledges several complications.

While there was no main effect of Definite Number in this model, there was an interaction between Indefinite Location and Definite Number. In the case of a subject indefinite, more subject completions were observed when the object noun was singular (10d) than plural (10c); in the case of an object indefinite, more subject completions were elicited when the subject noun was plural than when it was singular (10b) than plural (10a), *z* = 3.67, *p* < 0.001. This reversal is to be expected if the indefinite provides the preferred candidate for the correlate, but a plural distractor interferes with the distinctiveness of the indefinite target.

These patterns are consistent with the theoretically-motivated assumption that *which*-remnants in sluicing prefer the antecedent with the most accessible set of individuals. In this case, the indefinite description *some* makes a set of alternatives salient in the discourse, as opposed to a definite description, which arguably introduces a plural sum of individuals that can be interpreted as a single entity (Link, 1983). Accordingly, responses were transformed to reflect the pairing in which the remnant forms a contrast with the indefinite, as depicted in Table 3. The transformed response data was modeled as a logistic linear mixed effects regression model using the lme4 package (Bates and Maechler, 2009) with by-subjects and by-items random slopes and intercepts and deviation coding as before. The result is shown in Table 4.

**Table 3:** **Experiment 1: responses by the percentage of cases in which the indefinite was selected as the correlate**.

**Correlate choice**	**Plural interference**		**Correlate mean**	**Interference difference**
	**Interference**	**No interference**		
Object	72% (4)	81% (3)	76% (3)	9%
Subject	67% (4)	75% (4)	71% (3)	8%
Interference mean	69% (3)	78% (3)		

*Standard errors in parentheses*.

**Table 4:** **Experiment 1: results of linear mixed effects regression model on the proportion of transformed responses**.

	**Estimate**	**Std. error**	**Wald *Z***	***p*-estimate**
(Intercept)	1.661	0.285	5.835	< 0.001
Correlate location	−0.177	0.361	−0.49	0.624
Definite number	−**0.474**	**0.199**	−**2.38**	< **0.05**
Correlate × Definite number	−0.038	0.208	−0.181	0.857

*Significant effects are printed in bold*.

The model supports a sole effect of Definite Number, in that a plural definite noun (*the wines/the tourists*) resulted in fewer responses that co-referred with the indefinite target (*M* = 69%, *SE* = 3) than singular definite distractors (*M* = 78%, *SE* = 3), *t* = −2.38, *p* < 0.05, although the indefinite is still generally preferred.

#### Discussion

The results suggest that the structures are not fully ambiguous: there is a strong preference to associate the remnant with an indefinite correlate. The transformed results also show clear support for general similarity-based interference, in that plural definite distractors, which shared the plurality feature with an indefinite correlate, attracted more remnant resolutions than singular definite distractors.

## Experiment 2

A second experiment was conducted to test the central predictions outlined above. First, by *Locality*, subject position indefinite nouns should elicit an online processing penalty over their more local, object position counterparts. Second, by *Nominal Advantage, wh*-restrictors with a fully specified nominal should facilitate the retrieval of their correlates compared to pronominal restrictors like *ones* by virtue of providing a richer feature set for cue-matching. Lastly, by *Structure-Dependent Interference*, plural definite nouns should exert a greater interference effect on retrieval when occupying object position, and, further, that such effects should manifest predominantly when the cues for retrieval are poor.

### Method

#### Participants

Fifty-six native English speaking college students with normal or corrected-to-normal vision were recruited for the experiment, and were compensated $10 for participation. Nine students were excluded due to excessive blinks leading to extreme data loss, as detailed below, resulting in a final dataset of 47 subjects.

#### Materials

Twenty-four sextets were constructed from the items in Experiment 1, modified so that there were three animate subject correlate (12a–c) and three inanimate object correlate (12d–f) conditions. In both cases, there was a condition with a definite plural distractor and a fully-specified nominal in the *wh*-restrictor, e.g., *tourists* or *wines* (12a,d), a definite plural distractor and the plural pronoun *ones* (12b,e), and a definite singular distractor and the plural pronoun *ones* (12c,f). Note that in (12a–c) the indefinite noun appears in the object, and so by hypothesis the local and preferred, position. The pipe symbol “|” indicates how materials were later divided into seven regions for analysis. All conditions were identical after the remnant region

(12)
  - |The tourists |sampled |some wines, |but I've forgotten |which wines, …
  - |The tourists |sampled |some wines, |but I've forgotten |which ones, …
  - |The tourist |sampled |some wines, |but I've forgotten |which ones,
  - |Some tourists |sampled |the wines, |but I've forgotten |which tourists, …
  - |Some tourists |sampled |the wines, |but I've forgotten |which ones, …
  - |Some tourists |sampled |the wine, |but I've forgotten |which ones, …
      |since they all |seem the same to me.

Lexical level characteristics of length and frequency were computed for nouns in subject and object position. Subject (*M* = 6.83; *SE* = 0.27) and object nouns (*M* = 6.96, *SE* = 0.41) did not differ on number of characters, *t*_(23)_ = −0.24, *p* = 0.82. Two measures of frequency were obtained from the English Lexicon Project (Balota et al., 2007). Subject (*M* = 8.12; *SE* = 0.32) and object nouns (*M* = 8.86, *SE* = 0.42) did not differ on log HAL frequency, *t*_(23)_ = −1.56, *p* = 0.13. Further, subject (*M* = 2.26; *SE* = 0.15) and object nouns (*M* = 2.50, *SE* = 0.14) match on frequency calculated from SUBTLEX, *t*_(23)_ = −1.22, *p* = 0.23. In addition, the length of the remnant region was always included as a predictor in models of that region.

A reviewer notes that the spillover regions may not have been informative with respect to the intended interpretation. However, items were intentionally designed so that properties of the inner restrictor of the remnant provided the only disambiguating information. Further, spillover regions were consistent within an item across all conditions, and thus are unlikely to explain any effects. However, as noted above, it may be fruitful to explore the influence of the structure in unelided counterparts, as in *I don't know which ones (they sampled*/*were sampled*). Not all interesting contrasts could be presented in a single experiment, for fear of reducing statistical power or saturating readers with too many similar constructions. Another concern was that the example above contains the ambiguous pronoun *they* after the remnant. However, the item above is unique in that respect. As shown in the Appendix of Supplementary Material, no other item contains a pronoun of any sort.

#### Procedure

The experiment was presented using EyeTrack, the UMass Amherst presentation software (http://www.psych.umass.edu/eyelab/). Materials were presented in a sound isolated room on a 32-bit Dell Optiplex tower, running Windows 7, with peripheral programs and the Internet connection turned off. Text was presented as a single line in black 11pt monospaced font against a white background. The monitor was situated such that approximately three characters subtended 1° of visual angle. Eye movements were recorded on an SR Research Eyelink 1000 eye tracker, mounted on the table approximately 50 cm away from a 19” Mitsubishi Diamond Pro 900u flat-screen CRT monitor running at 170 Hz. Sampling rate was set to 1000 Hz. Drift correct was performed between each trial. Subjects were instructed to read naturally and for comprehension, and were encouraged to take breaks as often as needed.

All items were followed by comprehension questions probing the subject's interpretation (13). Questions were presented in CAPS to clearly differentiate comprehension questions from experimental materials.

(13) WHAT DID I FORGET?
  - Subject response: WHICH TOURIST(S)
  - *Object response:* WHICH WINE(S)

Subjects selected the answer from among two possible choices on a Microsoft USB Sidewinder gamepad. Question responses were not considered in the analysis below. Experiments lasted approximately 40 min on average.

#### Results

Individual trials were removed if the participant blinked once or more during the first pass on the remnant region. No trials were removed if blinks occurred in another region or during re-reading of the remnant. Individual trials were also removed if excessive blinking led to significant track loss, or if track loss occurred for some other reason during the experiment (< 4% of total trials).

Additionally, short (under 80 ms) and long (over 1200 ms) fixation times were removed from the data, as were trials with blinks on the remnant and track losses using the program EyeDoctor (http://www.psych.umass.edu/eyelab). Several standard eye tracking measures were used in the analysis (Rayner, 1998), computed with the DOS version of EyeDry analysis software: first *pass durations* (also known as gaze duration), the sum of all fixation durations within a region before leaving that region in any direction, *go past time*, the time spent after first entering a region to first moving past the region to the right, *percentage of regressions out* of and *percentage of regressions into* a region, second *pass time*, the time spent rereading a region once the region has been exited to the right including zero times indicating failure to re-read, and *total time*, the sum of all fixation times in a region during any point in reading (see, e.g., Staub and Rayner, 2007, for a concise review of these measures). Means and standard errors for these measures are presented in Table 5 below.

**Table 5:** **Experiment 2: means and standard deviations for all eye tracking measures**.

		**Subject**	**Verb**	**Object**	**But I don't know**	**Which X**	**Spill over**	**Final region**
		**FIRST PASS**						
Interference nominal	Object	263 (15)	319 (13)	384 (16)	368 (14)	281 (9)	325 (13)	391 (18)
	Subject	292 (16)	320 (18)	327 (14)	375 (16)	300 (10)	335 (14)	405 (23)
Interference pronoun	Object	282 (16)	317 (14)	372 (15)	373 (15)	230 (8)	336 (14)	394 (18)
	Subject	298 (16)	292 (14)	318 (15)	352 (14)	257 (7)	338 (15)	393 (20)
No interference pronoun	Object	282 (21)	336 (17)	366 (14)	368 (15)	234 (8)	333 (15)	408 (20)
	Subject	295 (16)	321 (15)	298 (15)	370 (15)	264 (9)	339 (14)	418 (22)
		**GO PAST**						
Interference nominal	Object	263 (15)	414 (18)	502 (23)	385 (15)	338 (15)	387 (29)	818 (66)
	Subject	326 (29)	380 (21)	438 (25)	389 (17)	363 (15)	350 (19)	834 (54)
Interference pronoun	Object	282 (16)	381 (18)	527 (29)	392 (17)	267 (14)	352 (17)	854 (66)
	Subject	298 (16)	402 (29)	472 (30)	374 (17)	303 (18)	352 (16)	1071 (94)
No interference pronoun	Object	282 (21)	464 (25)	559 (34)	405 (21)	279 (16)	377 (24)	908 (64)
	Subject	295 (16)	378 (20)	448 (29)	394 (18)	317 (20)	400 (25)	975 (70)
		**SECOND PASS**						
Interference nominal	Object	90 (12)	101 (16)	39 (9)	99 (18)	55 (11)	129 (17)	NA
	Subject	84 (12)	104 (17)	29 (7)	79 (13)	42 (11)	140 (16)	NA
Interference pronoun	Object	79 (12)	116 (15)	64 (13)	56 (12)	28 (7)	122 (18)	NA
	Subject	149 (21)	173 (21)	84 (16)	88 (16)	61 (16)	151 (19)	NA
No interference pronoun	Object	128 (14)	154 (23)	73 (14)	68 (12)	37 (8)	123 (15)	NA
	Subject	110 (16)	137 (17)	87 (15)	87 (16)	45 (10)	148 (16)	NA
		**TOTAL TIMES**						
Interference nominal	Object	249 (17)	439 (23)	446 (19)	458 (23)	344 (16)	431 (20)	473 (23)
	Subject	323 (27)	408 (25)	365 (17)	449 (20)	353 (16)	436 (22)	504 (26)
Interference pronoun	Object	270 (18)	429 (23)	472 (23)	412 (20)	253 (12)	438 (23)	496 (25)
	Subject	361 (24)	473 (29)	425 (25)	439 (21)	311 (17)	466 (22)	522 (29)
No interference pronoun	Object	277 (20)	521 (32)	496 (27)	441 (20)	263 (13)	424 (20)	505 (24)
	Subject	328 (23)	449 (23)	411 (24)	451 (23)	307 (15)	465 (20)	546 (25)
		**REGRESSIONS OUT**						
Interference nominal	Object	NA	18 (3)	24 (3)	2 (1)	15 (3)	5 (2)	42 (4)
	Subject	NA	12 (3)	20 (3)	2 (1)	12 (3)	1 (1)	44 (4)
Interference pronoun	Object	NA	13 (3)	26 (3)	2 (1)	7 (2)	2 (1)	45 (4)
	Subject	NA	17 (3)	26 (3)	2 (1)	7 (2)	2 (1)	49 (4)
No interference pronoun	Object	NA	23 (3)	24 (3)	3 (1)	10 (2)	5 (2)	47 (4)
	Subject	NA	14 (3)	25 (3)	3 (1)	11 (2)	5 (2)	52 (4)
		**REGRESSIONS IN**						
Interference nominal	Object	68 (6)	32 (4)	5 (2)	22 (3)	8 (2)	35 (4)	NA
	Subject	54 (6)	29 (4)	4 (2)	16 (3)	4 (1)	37 (4)	NA
Interference pronoun	Object	62 (6)	35 (4)	7 (2)	12 (3)	3 (1)	36 (4)	NA
	Subject	66 (5)	38 (4)	11 (2)	13 (3)	4 (2)	36 (4)	NA
No interference pronoun	Object	78 (4)	34 (4)	10 (2)	16 (3)	4 (2)	36 (4)	NA
	Subject	60 (5)	34 (4)	13 (3)	18 (3)	5 (2)	42 (4)	NA

Linear mixed effects regression models were used for all statistical analyses. Fixation and reading time measures (first pass, go past, second pass, and total times) were analyzed with linear regression models, whereas proportion data (regressions in and out of a region) were analyzed with logistic regression models. As models with maximal random effects error structures (as recommended by Barr et al., 2013) typically failed to converge, all models reported here were specified with by-subject and by-items random intercepts, but not with random slopes. Fixed effect predictor contrasts were assigned deviation coding that best cohered with the conceptual aims of the study. To assess the presence of a Locality bias, object noun correlates were coded as the baseline for the Correlate position predictor. To evaluate the effect of Interference, cue-poor probes (*which ones*) without plural interference were treated as the baseline for the Interference predictor, so that the model tests for similarity-based interference effects for nominal and pronominal cues over a no interference condition with a pronominal cue.

Instead of reporting the statistical results of each measure individually, the results are discussed in terms of the predictions of interest, noting when other effects were present. All significant effects for the measures of interest are reported.

#### Locality

Evidence for the Locality bias was observed in multiple measures of the eye movement record. The earliest evidence was found in first pass times immediately at the remnant (Region 5). indefinite subject correlates (*M* = 274 ms, *SE* = 5) elicited longer first pass times than indefinite object correlates (*M* = 249 ms, *SE* = 5), *t* = 3.88. No other effects were observed in first pass times. Additional evidence for the Locality bias appears in later eye movement measures, as well. Indefinite subject correlates elicited longer go past times than object correlates on the remnant region (*M*_*Subject*_ = 328 ms, *SE* = 10; *M*_*Object*_ = 295 ms, *SE* = 9), *t* = 2.72, and on the sentence final region, (*M*_*Subject*_ = 960 ms, *SE* = 43; *M*_*Object*_ = 860 ms, *SE* = 38), *t* = 2.12. Fixed effects of models substantiating the above effects are provided in Table 6.

**Table 6:** **Experiment 2: linear mixed effects regression models for first fixation and go past times on Remnant and go past times on the sentence final region**.

	**First pass times on remnant**			**Go past times on remnant**			**Go past times on final region**		
	**Estimate**	**Std. error**	***t*-value**	**Estimate**	**Std. error**	***t*-value**	**Estimate**	**Std. error**	***t*-value**
(Intercept)	231.863	18.748	12.368	266.994	35.923	7.432	899.026	80.558	11.16
Interference nominal	−7.037	5.745	−1.225	22.199	15.588	1.424	−**83.699**	**34.589**	−**2.42**
Interference pronoun	**18.517**	**8.139**	**2.275**	−18.053	10.95	−1.649	54.613	41.688	1.31
Locality	**12.827**	**3.303**	**3.883**	**17.02**	**6.254**	**2.721**	**53.623**	**25.251**	**2.124**
Length	5.502	3.453	1.593	8.485	6.644	1.277	NA	NA	NA
Interference nominal × Locality	1.357	4.674	0.29	−2.685	8.812	−0.305	−48.508	40.662	−1.193
Interference pronoun × Locality	−2.378	4.653	−0.511	1.398	8.865	0.158	58.372	45.79	1.275

*Effects with t-values above |2| are shown in bold*.

**Figure 1:**
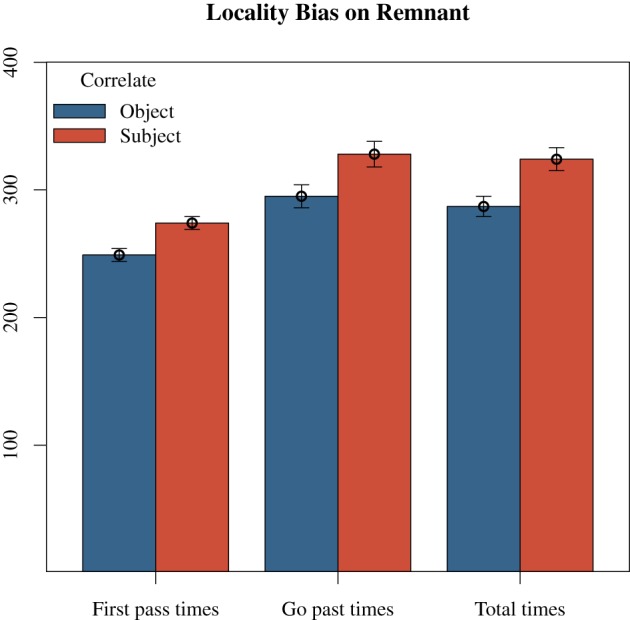
**Experiment 2: effect of Locality on the remnant region**. Times presented in ms.

Further, violating Locality manifested in a persistent penalty for total times, as indefinite subject correlates were significantly longer in the sentential subject region (*M*_*Subject*_ = 337 ms, *SE* = 40; *M*_*Object*_ = 265, *SE* = 11), *t* = 4.65, the remnant (*M*_*Subject*_ = 324, *SE* = 9; *M*_*Object*_ = 287, *SE* = 8), *t* = 3.40, and the final region (*M*_*Subject*_ = 524 ms, *SE* = 15; *M*_*Object*_ = 491 ms, *SE* = 14), *t* = 2.20. Models computed for total times are provided in Table 7. In addition, there was a marginally significant penalty for indefinite subject correlates (*M* = 147 ms, *SE* = 10) compared to indefinite object correlates (*M* = 125, *SE* = 10) on the spill-over region in second pass re-reading times, *t* = 1.95. A summary of the main effects on the remnant is provided in Figure 1.

**Table 7:** **Experiment 2: linear mixed effects regression models for total times on the sentence initial, remnant, and sentence final regions**.

	**Total times on subject region**			**Total times on remnant**			**Total times final region**		
	**Estimate**	**Std. error**	***t*-value**	**Estimate**	**Std. error**	***t*-value**	**Estimate**	**Std. error**	***t*-value**
(Intercept)	301.190	24.644	12.222	257.591	32.441	7.940	503.846	36.847	13.674
Interference nominal	−15.269	11.066	−1.380	23.878	14.036	1.701	−20.769	11.479	−1.809
Interference pronominal	13.851	11.069	1.251	−12.632	9.842	−1.284	5.633	11.483	0.491
Locality	**36.310**	**7.816**	**4.646**	**19.117**	**5.620**	**3.402**	**17.834**	**8.107**	**2.200**
Length	NA	NA	NA	9.412	5.996	1.570	NA	NA	NA
Interference nominal × Locality	−2.205	11.079	−0.199	−14.451	7.968	−1.814	0.348	11.494	0.030
Interference pronominal × Locality	12.028	11.071	1.086	10.805	7.958	1.358	−3.258	11.485	−0.284

*Effects with t-values above |2| are shown in bold*.

However, a few measures showed a cost for indefinite object correlates. There were more regressions into the sentential subject region for items with indefinite object correlates (*M* = 70%, *SE* = 3) than indefinite subject correlates (*M* = 60%, *SE* = 3), *t* = 2.64, *p* < 0.01. The increased rate of regressions into the subject region may correspond to increased global re-reading for indefinite object correlates, as opposed to regressing back into a specific region. In addition, indefinite object correlates elicited longer total times than indefinite subject correlates did in the region containing the object noun (*M*_*Object*_ = 471 ms, *SE* = 13; *M*_*Subject*_ = 401 ms, *SE* = 13), *t* = −4.73. This effect could be explained if total times corresponded to additional re-reading of the correlate. However, it is unclear whether such an explanation can be strongly maintained without supporting evidence from regressions in and second-pass reading measures, of which there is little evidence.

#### Nominal advantage

For the second prediction, we expect that nominal restrictors (*which wines/tourists*) should receive a processing benefit over pronominal restrictors (*which ones*), due to greater specificity of the retrieval cue (also known as “cue diagnosticity”; see Martin and McElree, 2009, 2011; Van Dyke, 2011). Indeed, we find the expected advantage for nominal probes in a variety of measures. In go past times, there was a 117 ms advantage for nominal restrictors over pronominal ones on the final region, *t* = −2.42; see the *Interference nominal* row in Table 6. There were fewer regressions into the object region when a nominal cue in the remnant followed (*M* = 5%, *SE* = 1) as compared to a pronominal cue in the restrictor (*M* = 11%, *SE* = 2), *t* = −2.93, *p* < 0.01.

**Figure 2:**
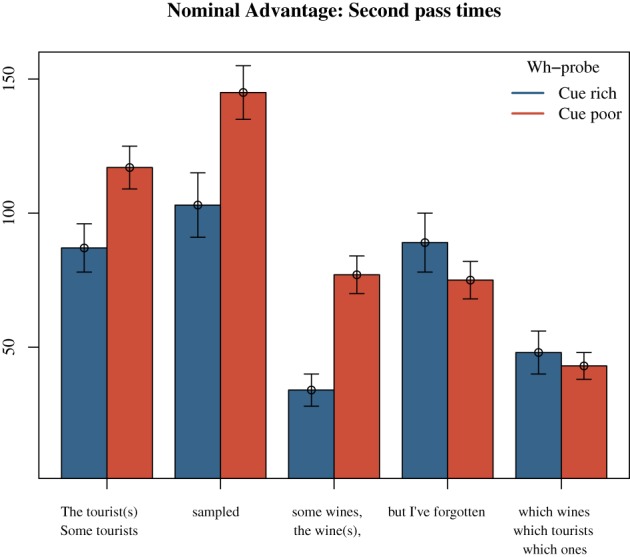
**Experiment 2: effect of Locality on the remnant region**. Nominal advantage in second pass re-reading times. Times presented in ms.

Further, the advantage for nominal cue conditions was considerable in second pass re-reading times of every region of the matrix clause. Nominal restrictors elicited shorter second pass times in the subject region (*M*_*Nominal*_ = 87, *SE* = 9; *M*_*Pronominal*_ = 119, *SE* = 11), *t* = −2.61, the verb region (*M*_*Nominal*_ = 103, *SE* = 12; *M*_*Pronominal*_ = 145, *SE* = 14), *t* = −3.21, and the object region (*M*_*Nominal*_ = 34, *SE* = 6; *M*_*Pronominal*_ = 80, *SE* = 10), *t* = −4.31; see Figure 2. There were also shorter total times for nominal probes in total times for the subject region (*M*_*Nominal*_ = 423 ms, *SE* = 17; *M*_*Pronominal*_ = 485 ms, *SE* = 20), *t* = −2.60.

One region witnessed effects other than were expected: there were more regressions into Region 4 for Nominal than Pronominal conditions (*M*_*Nominal*_ = 19%, *SE* = 2; *M*_*Pronominal*_ = 15%, *SE* = 1), *t* = −2.28, *p* < 0.05. There is no ready explanation of this small effect, as the content of the region was identical in all conditions.

#### Structure-dependent interference

The central prediction of an interaction between similarity-based interference and structural position of the correlate was supported by later eye movement measures. Importantly, a penalty was predicted only for pronominal probes in the remnant like *ones*, but not when the cue was fully specified, as in the case of nominal probes. As expected, there was a greater penalty for second pass times for definite plural object nouns (*the wines*) and a subject correlate (*some tourists*) when the *wh*-restrictor was a pronominal. In the subject region, the penalty for conditions with pronominal restrictors was significantly greater for indefinite subject correlates (*d* = 70 ms) than for indefinite object correlates (*d* = −18 ms), *t* = 3.54. Similar effects obtained in the verb region, with a 57 ms penalty for subject correlates over object correlates (*d* = −17 ms), *t* = 2.26. Both of these effects are shown in the final row in Table 8.

**Table 8:** **Experiment 2: nominal advantage in second pass re-reading times**.

	**Second pass on subject region**			**Second pass on verb region**			**Second pass on object region**		
	**Estimate**	**Std. error**	***t*-value**	**Estimate**	**Std. error**	***t*-value**	**Estimate**	**Std. error**	***t*-value**
(Intercept)	107.007	13.958	7.666	131.059	21.721	6.034	63.048	11.175	5.642
Interference nominal	**−20.473**	**7.849**	**−2.608**	**−29.972**	**9.345**	**−3.207**	**−29.309**	**6.794**	**−4.314**
Interference pronominal	7.929	7.849	1.01	15.963	9.348	1.708	11.965	6.794	1.761
Locality	8.137	5.543	1.468	7.064	6.6	1.07	4.291	4.797	0.894
Interference nominal × Locality	−12.287	7.856	−1.564	−7.381	9.357	−0.789	−10.67	6.8	−1.569
Interference pronominal × Locality	**27.778**	**7.851**	**3.538**	**21.11**	**9.349**	**2.258**	7.044	6.795	1.037

*Effects with t-values above |2| are shown in bold*.

**Figure 3:**
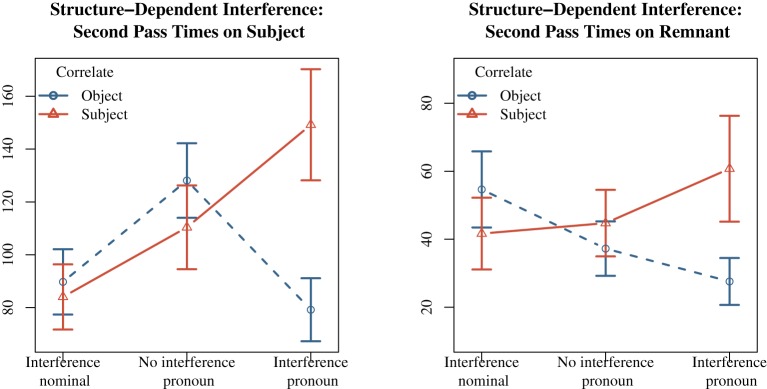
**Experiment 2: Structure-Dependent Interference effects on subject and remnant regions**. Times presented in ms.

On the remnant region, there was again a greater penalty for indefinite subject correlates (*d* = 33) than for indefinite object correlates (*d* = 8), *t* = 2.11; see Table 9 and Figure 3. What's more, nominal restrictors showed a small 13 ms second pass time advantage for indefinite subject correlates and plural distractor objects, *t* = −2.08 in second pass times; Table 9. Finally, in the verb region, there was a greater total times penalty for indefinite subject correlates and plural distractors with pronominal retrieval cues (*d* = 72 ms) than the no interference baseline (*d* = 44 ms), *t* = 2.62.

**Table 9:** **Experiment 2: Structure-Dependent Interference effects in second pass re-reading times on the remnant region**.

	**Second pass times on remnant region**		
	**Estimate**	**Std. Error**	***t*-value**
(Intercept)	31.428	23.059	1.363
Interference nominal	−1.576	10.212	−0.154
Interference pronominal	2.453	7.186	0.341
Locality	4.722	4.121	1.146
Length	2.58	4.347	0.594
Interference nominal × Locality	−**12.126**	**5.843**	−**2.075**
Interference pronominal × Locality	**12.313**	**5.836**	**2.110**

*Effects with t-values above |2| are shown in bold*.

#### Discussion

Results from the reading experiment support all three predictions of interest. There was early and sustained support for violating the Locality bias, and an advantage for Nominal cues that manifested in go past and second pass measures. These two effects interacted with respect to interference and cue-specificity: there were greater interference effects when the distractor occupied the preferred object position, such that the effect was enhanced when cues for retrieval at the remnant were partial. The results are thus compatible with previous findings of retroactive interference (e.g., Van Dyke and McElree, 2006; Van Dyke and Johns, 2012), but also adds support to the growing body of evidence that retrieval is modulated by the position of the antecedents (e.g., Van Dyke and McElree, 2011; Chow et al., 2014; Dillon et al., 2014; Kush and Phillips, 2014, among others). While the results are clearly consistent with the central predictions of a cue-based parsing system in which the location of targets in the sentence is not ignored during retrieval, the issue of how precisely to utilize such information within cue-based parsing models is far from settled. I return to this question the General Discussion.

As noted by a reviewer, evidence for the central predictions manifested at somewhat different time courses, although we should exercise caution when assigning linking assumptions to eye movement measures (Clifton et al., 2007). Whereas, evidence for Locality and Nominal Advantage appeared in various measures, support for Structure-Dependent Interference was only observed in the “late” measure of second-pass times, as subjects re-read portions of the sentence. This delayed effect is compatible with multiple interpretations, including a multiple stage model of anaphoric processing in which measures occurring later in the eye movement record could reflect processing at a secondary stage of discourse integration, perhaps along the lines of Garrod and Sanford's (1990, 1994) bonding and resolution model. If this were the case, Structure-Dependent Interference might reflect difficulty interpreting the link between a poorly specified remnant and a correlate, rather than retrieval difficulty. Alternatively, that the effect appears relatively late in the eye movement record could be attributed to a lag resulting from poor quality matches. In this case, the integration difficulty would directly reflect increased interference from distractors in structurally preferred positions. The results do not arbitrate between these, or any number of, additional possibilities, which must instead be resolved through careful experimental design.

A reviewer proposed that several of the sentences in (12) are ambiguous, in that *which ones* may also co-refer with definite plural nouns. The above design depended on the assumption that definite nouns fail to provide an appropriate antecedent, as discussed in connection with examples (5–6). Yet, there may be a few systematic exceptions to the generalization that the remnant cannot correspond to a definite correlate. Discussion in the literature centers around contrasts like (13) below, which shows that a d-linked *which* remnant can take a definite noun as a correlate, but a simple *wh*-phrase like *what* cannot (Chung et al., 1995; Dayal and Schwarzschild, 2010).

(13)
  - John announced he had eaten the asparagus. We didn't know which asparagus.
  ^*^ John announced he had eaten the asparagus. We didn't know what.

These cases have been given various analyses. Chung et al. (1995) suggest that definite nouns are available as correlates whenever they are compatible with the pragmatic contribution of the remnant. They attribute the contrast in (13) to an intuitive conflict in familiarity between the definite *the asparagus* and the novelty imposed by *what* in (13b), a conflict that (13a) avoids.

In contrast, Dayal and Schwarzschild (2010) identify several cases in which presumed speaker knowledge, rather than familiarity, is the distinguishing factor. In their account, (14a) is infelicitous because the speaker has contradicted herself: the knowledge state that permits the speaker to assert that John talked to the detective places the speaker in a sufficient epistemic position to answer the question embedded under the sluice, i.e., *which detective did he talk to*? The corresponding assertion with an indefinite (14b) does not place the speaker in such a specific knowledge state as to warrant a self-contradiction (though see Barker, 2013; Barros, 2013, for recent commentary).

(14)
  ^*^ John talked to the detective. I don't know which detective (he talked to).
  - John talked to a detective. I don't know which detective (he talked to).

They also observe that a definite correlate is sometimes available when it does not carry a uniqueness presupposition, as illustrated by the examples in (15) where there is no requirement that there is a singular, identifiable train or particular hospital in the context (akin to so-called weak definites, e.g., Carlson and Sussman, 2005; Aguilar Guevara, 2014).

(15)
  - John is going to take the train, but he doesn't know yet which train (he is going to take).
  - They took him to the hospital. She wouldn't tell us which hospital (they took him to).

As would be predicted, a definite that corresponds to a unique individual within a given context, as in *the Chief of Police* in (16), cannot serve a correlate to the remnant.

(16) ^*^Ed reported the matter to the Chief of Police but Joe couldn't figure out which chief of police (he reported the matter to).

Although it is unclear whether these examples are as acceptable when fully elided, e.g., *John is going to take the train, but he doesn't know which*, the experimental items were reviewed to determine whether the definite noun could sensibly be interpreted as a correlate to the remnant^4^. Two possible types of cases were observed. The first case involved collective nouns whose members could perform an action on behalf of others in the group (the ? mark reflects my judgment that these sentences are somewhat degraded):

(17)
  - ? The trustees donated 10 million to the university, but I don't know which ones.
  - ? The professors wrote a letter to the dean, but it doesn't matter which ones.

For example, (17a) could be interpreted so that some of the trustees but not others were responsible for the donation. Here, the definite *the trustees* is interpreted as a collective entity, in which the donation was performed on behalf of the entire group. Although not many items in the experiment permit such a reading, one contender is *The nurses threatened to strike over some contracts, but I'm not sure which ones*.

The second case was one in which the remnant *which ones* does not refer directly to the definite noun phrase. Instead, co-reference appears to be coerced through a partitive interpretation taking the plural definite as the maximal set in order to derive a salient subset (a refset) from it (see Moxey and Sanford, 1993, for terminology). For example, such a reading might paraphrase (12d) as *Some tourists sampled the wines, but I don't know which (ones) of the wines they sampled*. The coercion process could posit a silent or elided partitive phrase, as proposed for bare determiner phrases like *Many (of them) sat down* (Gagnon, 2013). Again, very few plausible cases were found in the experimental items. Two possible cases include the example used as illustration throughout the paper (12), and *Some workers loaded the trucks, but I'm not certain which ones*. Such cases are perhaps strengthened by a distributive semantics of the verb, e.g., a sampling wine involves trying some, but not all, of it.

To assess empirically whether ambiguity could explain the effects observed above, I conducted a *post-hoc* by-items analysis of the results from Experiment 1. Averaging across conditions, no item was biased toward the definite noun completion. However, splitting the data by position of the indefinite revealed that eight items were either biased toward (definite subject: 13, 18, 21; definite object: 12, 24) or on par with (object definite: 2, 10, 18) the indefinite as a correlate. For most measures, there were no differences in the overall statistical effects when these items were removed^5^. However, removing potentially ambiguous items did weaken the interaction between Locality and Interference in second pass times: although the penalty for non-local correlates was still significantly greater for remnants with pronominal restrictors in the sentence-initial region, the interaction did not persist in following region, even though the interaction was still apparent in other measures, including total times. Thus, even though a plausible definite distractor could have engendered a longer lasting interference penalty from the indefinite, it is unlikely to be the primarily source of the effects reported here.

As a final note, ambiguity only becomes a genuine confound if it could otherwise explain the effects attributed to another variable. The only sentences that could have been ambiguous are those with cue-poor (*which ones*) remnants and plural definite distractors, i.e., (12b, d). Several other studies of pronominal ambiguity suggest that competing interpretations do not always result in processing penalties (e.g., attachment ambiguity explored in van Gompel et al., 2001, 2005), especially cases involving pronouns (e.g., Greene et al., 1992). Indeed, in the present case of sluicing, Frazier and Clifton (1998) report that ambiguity between subject and object position correlates did not slow readers down, provided that there was an indefinite correlate in the preferred, object position, like *someone* in (8a). Therefore, it is not yet clear how ambiguity would explain the effects I hope to attribute to interference.

Additionally, although the possibility of ambiguity might challenge whether we can truly interpret the effect of a plural definite as interference *per se*, it cannot fully account for the interaction between the plurality of the definite and its structural location. That is, irrespective of whether or not a definite noun is a possible correlate to *which* remnants, ambiguity does not explain why a plural definite in object position would elicit greater reading penalties than in subject position. Nevertheless, potential ambiguities could be more tightly controlled or even exploited (as in Harris, 2013, 2015) in future studies.

## General discussion

The central question explored in the experiment above was whether positional information modulates similarity-based interference effects in sluicing structures. There was clear evidence that it does. The central manipulation capitalized on the unique syntactic properties of sluices in two ways. First, the Locality bias was employed to impose a preference for structural position of the correlate to a remnant in the elided clause. Second, the lexical content of the inner restrictor of the remnant was manipulated to examine the role of cue-strength in retrieval.

As previously mentioned, this study is not the first to exploit sluiced sentences in an argument in favor of content-addressable retrieval systems. Martin and McElree (2011) utilized two main properties of an object position correlate in sluiced sentences like (14) in SAT and eye tracking. The correlate appeared on its own or within a conjunct in object position, and when the correlate was contained within a conjunct, which position of the conjunct it occupied (first or second conjunct position). The verbal syntax of only one member of the conjunct, *typed (something)*, provides a correlate, which varied according to whether the object was overt or not, to associate with the remnant *what*.

(14)
  - Michael (slept and) studied (something), but he didn't tell me what_1_ <he typed t_1_>.
  - Michael studied (something) (and slept), but didn't tell me what_1_ <he typed t_1_>.

The design varied the distance between the correlate and remnant, along with the size of the elided material that was to be recovered. In keeping with the findings above, they found that readers spent longer re-reading distant antecedents (14b) than local ones (14a), and suggested, as I have, that interfering antecedents degrades the quality of a match with potential antecedents in memory. However, the materials of their study are quite different from the ones above in three respects. First, as only one conjunct provided a proper correlate (which sometimes had to be sprouted) to the remnant, the experiment lacks the conditions for fully investigating similarity-based interference from other noun phrase distractors. Second, the correlate was always in the object position, thereby satisfying the Locality bias, at least in a broad sense. Third, while the remnant varied according to *wh*-element type (*what, which*, and *where*), they did not manipulate the properties of the inner restrictor of the remnant to provide explicit cues to guide the dependency formation. The study above therefore contributes very different, yet congruent, evidence in favor of interference effects in retrieving correlates for sluiced sentences.

It is worth comparing Martin and McElree's study to the present one for another reason, as well. They found that the presence of a conjunct over a single noun in object position did not affect retrieval in either reading time or a SAT task, and concluded that retrieval processes access the material for ellipsis directly on the basis of its content via a cost-free pointer mechanism, in line with studies on verb phrase ellipsis (Frazier and Clifton, 2001, 2005; Martin and McElree, 2008, 2009). However, it is possible that the mechanisms responsible for retrieving a correlate for the remnant are distinct from those responsible for recovering the elided IP material. Given the previously discussed dependency between resolving the remnant and determining the appropriate syntax of the ellipsis, it stands to reason that the former might be prioritized over the later, rather than attempting to solve two retrieval problems at once. Although it is theoretically possible that the ellipsis site lacks an explicit syntactic representation (Chung et al., 1995), there is good evidence for syntactic structure in sluicing ellipsis from both theoretical (e.g., Merchant, 2001; van Craenenbroeck, 2010) and experimental (e.g., Frazier and Clifton, 2001, 2005; Poirier et al., 2010) literature, in which case retrieving the ellipsis site is unlikely to reduce to simply pairing a correlate to the remnant of ellipsis.

Finally, one might be concerned that increased temporal distance, and thus decay, between the subject and the remnant might sufficiently explain the Locality bias, thereby eliminating structural information *per se* as a factor in the retrieval process. However, this explanation is unlikely given the results of Poirier et al.'s (2010) cross-modal priming study, in which printed targets related to the subject (*the handyman*) and dative object (*the programmer*) distractors were presented at two probe points in an auditory sentence: immediately after the offset of the remnant *_1_ or 500 ms downstream *_2_.

(15) The handyman threw a book to the programmer but I don't know which book *_1_ and no one *_2_ else seems to know.

There was no difference between decision times for targets related to subject and dative object nouns until position *_2_ (which showed a priming effect for the object), suggesting that subject and the dative object nouns were equally active at the remnant of the ellipsis. Crucially, these effects do not contradict the results of the reading study, since probes related to the indefinite target *a book* could not be tested, given that they were repeated in the inner restrictor of the *wh*-phrase *which book*. If the restrictor were replaced with a cue-poor probe like *which ones*, we would expect an advantage for more local antecedents at, or soon after, the remnant.

Several models of sentence processing could in principle accommodate the findings reported above, models which diverge on how to account for the differences observed between subject and object position correlates. Naturally, the results of a single study cannot determine whether the effect of position reflects temporal precedence, linear distance, or, as I have suggested, structural information. Although various interpretations are possible, structural information has been shown independently to impact the earliest stages of retrieval in several related domains. It stands to reason that retrieval might privilege items located in preferred structural positions, even when the preference is not grammatically controlled. Of course, the nature of the mechanisms that underlie this putative advantage will remain unsettled until an effect of structural privilege is replicated in a design that dissociates structure from other factors, like linear order. Fortunately, sluicing ellipsis offers just the right sort of flexibility to tease such issues apart in the future.

Moreover, uncovering how the processor resolves the multiple dependencies required for interpreting sluiced sentences has only just begun. The configurational possibilities of sluicing ellipsis provide a rich testing ground for disentangling the retrieval processes that are charged with recovering linguistic antecedents and integrating them into a representation as it unfolds during real-time comprehension. While numerous questions remain, one major challenge is the stage at which semantic and discourse information informs dependency formation in ellipsis, and whether information structural cues or strongly biased contexts can favor potential antecedents in the same way that structural information can. At the minimum, the present study provides additional support for converging evidence for cue-based parsing, and that the mechanisms underlying such retrieval are not wholly blind to the structural location of products in memory.

## Funding

The author gratefully acknowledges financial support from Pomona College.

### Conflict of interest statement

The author declares that the research was conducted in the absence of any commercial or financial relationships that could be construed as a potential conflict of interest.

## Footnotes

^1^Fully ambiguous sentences also show a strong Locality bias in silent reading, as explored in the controls for a different study (Harris, 2015). Subjects saw four ambiguous sluiced sentences like “An editor called a journalist, but I can't say which one it was any more” and were asked how they interpreted the sentences. There was an 86% bias toward the most local, object-correlate interpretation. In addition, the vast majority of the subjects (*N* = 48) displayed a complete or majority Locality bias for the items: 3 subjects were at chance, and only 1 subject had a consistent preference for non-local correlates.

^2^Frazier and Clifton (2005) argued that this asymmetry indicates the presence of syntactic structure within the ellipsis site, as the restriction on accessibility follows from their “conjunction domain hypothesis,” an independently motivated syntactic constraint on extraction. See Martin and McElree (2011) for commentary.

^3^The dependency between retrieval of a correlate and the syntax of the ellipsis site raises a number of pertinent issues regarding the relationship between the Locality Bias and parallelism between the matrix and the second conjunct. The interpretation of sluicing is known to be sensitive to parallelism (Carlson, 2002; Dickey and Bunger, 2011), as is conjunction generally (Frazier et al., 1984). If the processor takes the remnant phrase to be an object, perhaps on the basis Minimal Attachment (Frazier, 1978), the processor might prefer an object correlate to create structural parallelism between clauses. In an unpublished auditory forced-choice completion study (*N* = 48), Katy Carlson and I manipulated the surface position of the remnant to appear either as a subject (*A waiter talked to a guest, but …which waiter/which guest isn't clear*) or as an object (*A waiter talked to a guest, but …it's not clear which waiter / which guest*), along with the location of a pitch accent (subject or object). In addition to a general 66% bias for local object-position correlates, we found that subject position remnants (*which guest / which waiter isn't clear*) failed to elicit more subject responses than object position remnants did, and that pitch accent placement was the primary determinant of continuation choice. Further, accenting the object resulted in 8% more object continuations with a surface subject remnant, suggesting that linguistic focus (manifested here in the form of pitch accent), not syntactic parallelism, is the driving force behind the bias for local correlates in standard sluicing constructions.

^4^Thanks to Colin Phillips for discussion of this issue and for providing some of the examples that appear in this section.

^5^Other changes were that the slowdown on the Interference pronoun condition in first pass times was marginal, and the penalty for violating the Locality Bias disappeared for go past and total times in the final region, as well as for second pass times in the spill over region. Several other effects were significant once potentially ambiguous items were removed, including an interaction supporting Structure-Dependent Interference in the verb region on second pass times, a slowdown for nominal restrictors in go past times at the remnant, and a previously marginal advantage for nominal restrictors reached significance for total times in the sentence-final region.
