# Supplementary material for: Structure Modulates Similarity-Based Interference in Sluicing: An Eye Tracking study
Source: Front Psychol. 2015 Dec 18;6:1839. doi: 10.3389/fpsyg.2015.01839 (PMC4683205; doi:10.3389/fpsyg.2015.01839)
Supplement: Supplementary file 1 [file DataSheet1.DOCX]

**Appendix A**

*Experiment 1*. The complete 2x2 design is provided only for item 1. Subject correlates (1a,b) were crossed with object correlates (1c-d). Items with plural distractors (1a,c) were crossed with items with singular distractors (1b,d). Remaining items are presented only in case (1a), from which all other conditions may be derived.

1. a. Some waiters gathered near the gardens, but I don't know (which waiters / which gardens)

b. Some waiters gathered near the garden, but I don't know (which waiters / which garden)

c. The waiters gathered near some gardens, but I don't know (which waiters / which gardens)

d. The waiter gathered near some gardens, but I don't know (which waiter / which gardens)

2. Some tourists sampled the wines, but I've forgotten (which tourists / which wines)

3. Some lawyers debated the cases, but I can't say (which lawyers / which cases)

4. Some librarians lamented over the books, but it doesn't matter (which librarians / which books)

5. Some nurses are striking over the contracts, but I'm not sure (which nurses / which contracts)

6. Some art thieves stole the valuable pieces, but I'm not sure (which thieves / which pieces)

7. Some artists tried to copy the paintings, but I don't remember (which artists / which paintings)

8. Some farmers wanted to renovate the old barns, but I'm not certain (which farmers / which barns)

9. Some hackers uploaded the programs, but nobody knows (which hackers / which programs)

10. Some fans danced to the new songs, but I can't figure out (which fans / which songs)

11. Some students partied on the beaches, but I won't say (which students / which beaches)

12. Some bankers discussed the investments, but I don't know (which bankers / which investments)

13. Some movers damaged the antique vases, but it's not clear (which movers / which vases)

14. Some actors forgot the critical lines, but it doesn't matter (which actors / which lines)

15. Some mechanics repaired the classic cars, but I don't know (which mechanics / which cars)

16. Some hikers trekked through the mountains, but I don't know (which hikers / which mountains)

17. Some builders examined the skyscrapers, but I can't say (which builders / which skyscrapers)

18. Some boys vandalized the stores, but nobody is saying (which boys / which stores)

19. Some editors retracted the articles, but I don't know (which editors / which articles)

20. Some firemen saved the houses, but I'm not certain (which firemen / which houses)

21. Some loggers clearcut the forests, but nobody will say (which loggers / which forests)

22. Some rangers patrolled the parks, but it doesn't matter (which rangers / which parks)

23. Some workers loaded the trucks, but I'm not certain (which workers / which trucks)

24. Some spies recovered the documents, but nobody can reveal (which spies / which documents)

**Appendix B**

*Experiment 2*. The complete 3x2 design is provided only for item 1. Object correlates (1a-c) were crossed with subject correlates (1d-f). Items with plural distractors (1a,b,d,e) were crossed with items with singular distractors (1c,f). Remaining items are presented only in case (1a), from which all other conditions may be derived.

1. a. The waiters gathered near some gardens, but I don’t know which gardens, although I’m sure I could find out.

b. The waiters gathered near some gardens, but I don’t know which ones, although I’m sure I could find out.

c. The waiters gathered near some gardens, but I don’t know which ones, although I’m sure I could find out.

d. Some waiters gathered near the gardens, but I don’t know which waiters, although I’m sure I could find out.

e. Some waiters gathered near the gardens, but I don’t know which ones, although I’m sure I could find out.

f. Some waiters gathered near the garden, but I don’t know which ones, although I’m sure I could find out.

2. The tourists sampled some wines, but I’ve forgotten which tourists, since they all seem the same to me.

3. The lawyers argued about some cases, but I can’t say which lawyers, as I'm bound by a confidentiality agreement.

4. The librarians lamented about some books, but it doesn’t matter which librarians, because the library will be closing for good.

5. The nurses threatened to strike over some contracts, but I’m not sure which nurses, even though it’s an important cause.

6. The art thieves stole some valuable pieces, but I’m not sure which thieves, although I aim to find out.

7. The local artists tried to copy some rare paintings, but I don’t remember which artists, since I’m not part of the art world.

8. The farmers wanted to renovate some historic barns, but I’m not certain which farmers, despite thoroughly supporting the restoration.

9. The hackers uploaded some programs, but nobody knows which hackers, and it’s caused a lot of problems.

10. The fans danced to the new songs, but I can’t figure out which fans, since the video was badly damaged.

11. The students partied on some beaches, but I won’t say which students, to avoid getting into any more trouble.

12. The bankers discussed some investments, but I don’t remember which bankers, even though it’s a very important deal.

13. The movers damaged some expensive vases, but it’s not clear which movers, so our insurance will have to investigate.

14. The actors forgot some critical lines, but it doesn’t matter which actors, since the play has already been cancelled.

15. The mechanics repaired some classic cars, but I don’t know which mechanics, although I could probably ask around.

16. The hikers trekked through some mountains, but I don’t know which hikers, even if I admire the incredible journey.

17. The builders examined some skyscrapers, but I can’t say which builders, without consulting my boss first.

18. The boys vandalized some stores, but nobody is saying which boys, until the charges are formally filed.

19. The editors retracted some articles, but I don’t know which editors, though I expect it will be big news.

20. The firemen saved some houses, but I’m not certain which firemen, as it happened so very quickly.

21. The loggers clearcut some forests, but nobody will say which loggers, since it was a criminal offense.

22. The rangers patrolled some parks, but it doesn’t matter which rangers, if everyone stayed safe during the storm.

23. The workers loaded some trucks, but I’m not certain which workers, since it was so dark that night.

24. The spies recovered some stolen documents, but nobody can reveal which spies, for fear of causing another incident.
